# FOR BETTER OR FOR WORSE: MARITAL TRANSITIONS AND SEXUAL LIFE IN MIDDLE AND OLDER AGE

**DOI:** 10.1093/geroni/igz038.1116

**Published:** 2019-11-08

**Authors:** Jeffrey E Stokes, Elizabeth A Gallagher, Remona Kanyat, Cindy N Bui, Celeste Beaulieu

**Affiliations:** University of Massachusetts Boston, Boston, Massachusetts, United States

## Abstract

Marital transitions have known implications for health and well-being. However, little research has examined the effects of such transitions on adults’ sexual lives. This study uses longitudinal data from the National Study of Midlife Development in the United States (1995–2014) to compare different marital status and transition groups’ sexual activity, satisfaction, control, and effort throughout mid-and-later life. Across all outcomes, effects of marital status/transitions were contingent upon baseline values of the outcome. Consistently married adults reported more frequent sexual activity, greater sexual satisfaction, and greater effort put into sexual life than other groups when baseline values of those outcomes were average or above-average; such group differences were reduced or reversed at below-average baseline values. Among the not-married, women reported significantly less sexual activity than men. The consistently divorced/separated, consistently widowed, newly divorced/separated, and newly widowed all reported greater control over sexual life at follow-up than the consistently married, when baseline sexual control was average and/or below-average. Lastly, women reported lesser effort put into sexual life at follow-up than men across all groups, accounting for baseline effort; these gender gaps were least pronounced among the consistently and newly married, and most pronounced among the newly widowed and newly divorced/separated. Overall, findings indicate that implications of marital transitions for midlife and older adults’ sexual lives depend upon both gender and pre-transition context. Marriage is not always beneficial for sexual life; rather, poor quality sexual lives during marriage can reduce opportunities for improvement that may arise with marital transitions, including divorce and widowhood.

